# Lurasidone vs. Other Antipsychotics as Augmentation Strategies for Clozapine in Treatment-resistant Schizophrenia: An Observational Multicenter Prospective Study

**DOI:** 10.2174/011570159X399781250910115949

**Published:** 2025-10-08

**Authors:** Tiziano Prodi, Beatrice Benatti, Alessandro Grecchi, Margherita Oresti, Filippo Mazzoni, Giovanni Carnevale Miacca, Natascia Brondino, Bernardo Dell’Osso, Miriam Olivola

**Affiliations:** 1 Department of Mental Health, Department of Biomedical and Clinical Sciences “Luigi Sacco”, University of Milan, Milan, Italy;; 2 “Aldo Ravelli” Center for Nanotechnology and Neurostimulation, University of Milan, Milan, Italy;; 3 ASST Santi Paolo e Carlo Borromeo, Division of Psychiatry, Milan, Italy;; 4 Dipartimento di Salute Mentale e Delle Dipendenze, Ospedale San Paolo – Polo Universitario, ASST Santi Paolo e Carlo, Milan, Italy;; 5 Department of Brain and Behavioural Sciences, University of Pavia, Pavia, 27100, Italy;; 6 Dipartimento di Salute Mentale e Delle Dipendenze, ASST Fatebenefratelli-Sacco, Milan, Italy;; 7 Department of Psychiatry and Behavioral Sciences, Stanford University, Stanford, California, USA;; 8 Centro per lo Studio Dei Meccanismi Molecolari Alla Base Delle Patologie Neuro-psico-geriatriche”, University of Milan, Milan, Italy

**Keywords:** Lurasidone, clozapine, antipsychotic agents, Schizophrenia, treatment-resistant Schizophrenia, schizophrenia spectrum disorder

## Abstract

**Introduction:**

Clozapine, after its introduction, reshaped the landscape of Treatment-Resistant Schizophrenia (TRS) treatment, becoming the first-line treatment for that condition. However, many patients fail to respond to this drug alone. Clozapine-resistant schizophrenia (CRS) is associated with a more severe clinical presentation than TRS, manifesting in exacerbated symptoms and significantly diminished quality of life. The complex nature of CRS has prompted the development of augmentation strategies, which most commonly include another antipsychotic. The present multicenter observational study aimed to assess and compare the efficacy of Lurasidone augmentation alongside clozapine *versus* other second-generation antipsychotic combinations in patients with a schizophrenia spectrum disorder.

**Methods:**

A total of 45 patients with a diagnosis of a schizophrenia spectrum disorder and labeled as “treatment resistant” were included. Functional and psychometric assessments were made at the baseline, one month, and six months after the treatment. A linear mixed-effect regression was performed along with other appropriate statistical analyses.

**Results:**

A significant improvement over time was observed in the two groups for both the clinical and functional outcomes assessed, demonstrating the efficacy of a proper augmentation strategy in CRS management. Moreover, significantly lower psychiatric ward admissions were observed in the lurasidone group (*p<*.05).

**Discussion:**

Our findings suggest that lurasidone augmentation in CRS offers significant improvements in psychopathological domains similar to alternative augmentation strategies.

**Conclusion:**

Although further studies are needed to confirm our findings, lurasidone’s favorable side-effect profile should be considered.

## 1 INTRODUCTION

Schizophrenia is a complex and chronic psychiatric disorder, presenting significant challenges for both patients and clinicians due to its multifaceted symptomatology. While antipsychotic medications have demonstrated efficacy in managing symptoms for a majority of patients, a substantial subset continues to experience persistent symptoms despite receiving adequate trials of multiple antipsychotics [1]. This phenomenon is categorized as Treatment-Resistant Schizophrenia (TRS), a condition marked by the enduring presence of positive symptoms - such as hallucinations, delusions, and disorganized thinking - that severely impair functional capacity and quality of life [2]. Furthermore, negative symptoms, including social withdrawal and cognitive impairments, exacerbate the clinical complexity, underscoring the need for individualized, multimodal treatment strategies [3]. Current consensus defines TRS as the persistence of psychotic symptoms despite adequate adherence to at least two antipsychotic trials, typical or atypical, administered at therapeutic doses for a minimum duration of six weeks [1]. Epidemiological data estimate that up to 34% of individuals with schizophrenia meet the criteria for TRS, highlighting its substantial prevalence and clinical significance [4-6]. A similar rate of unresponsive or partially responsive cases is observed in other psychiatric conditions (such as Major Depressive Disorder), underlining that Treatment-Resistant conditions in psychiatry represent a major challenge needing more effective and tolerable treatments [7].

The impact of TRS extends well beyond the core symptomatology, significantly impairing psychosocial functioning, occupational prospects, and overall quality of life [8]. This multifaceted disorder poses substantial challenges to patient well-being, with many individuals facing limited social integration and reduced participation in daily activities. Compounding this issue, non-adherence to pharmacological regimens has been identified as a critical factor exacerbating the course of illness, further complicating the management and outcome of the condition [9]. These complexities underscore the urgent need for innovative and tailored therapeutic strategies, including integrating novel pharmacological treatments and psychological interventions. The clinical complexity of TRS requires a multifactorial approach, with a deep understanding of both the neurobiological underpinnings and psychosocial dimensions of the disorder to guide treatment selection and improve long-term outcomes [10].

Among the pharmacological interventions for TRS, clozapine stands as a cornerstone therapy. First introduced in the late 20^th^ century, clozapine significantly reshaped the landscape of TRS treatment [11]. Unique among second-generation antipsychotics, clozapine is characterized by its low D2 receptor occupancy, along with its action on the noradrenergic and glutamatergic systems, which distinguishes it from other medications in this class. Additionally, clozapine's antagonistic effects on alpha-adrenergic, muscarinic, and histaminergic receptors contribute to both its antipsychotic efficacy and its metabolic effects [12]. Despite its well-documented efficacy, clozapine's use is hindered by its potential for serious adverse effects, including agranulocytosis, which mandates routine hematological monitoring, and myocarditis, a rare but potentially fatal risk [13]. Nonetheless, clozapine's superior efficacy in alleviating both positive and negative symptoms of schizophrenia reinforces its indispensable role in the management of TRS. Pharmacokinetically, clozapine exhibits notable inter-individual variability. It is metabolized primarily by the liver, where active metabolites contribute to its therapeutic effects. A thorough understanding of clozapine's pharmacokinetics is essential for optimizing dosing strategies and minimizing the risk of adverse effects [14].

Despite the pivotal role of clozapine in treating TRS, between 40% and 70% of patients fail to respond to this drug alone. Clozapine-resistant schizophrenia could be described as the persistence of positive, negative, or cognitive symptoms with at least moderate severity, and less than 20% symptom reduction in patients with schizophrenia after an adequate clozapine trial [1, 15]. However, although a univocal definition of CRS is still lacking [1, 15-17], it has been reported that CRS is associated with a more severe clinical presentation than TRS, characterized by exacerbated symptoms and a significantly reduced quality of life [18]. Particularly, the different definitions of CRS are mainly based on positive symptoms, since the proposal to extend the definition to negative, cognitive, or functional symptoms has not been scientifically evaluated [15].

The complex nature of CRS has prompted the development of augmentation strategies, which have become integral adjuncts to traditional antipsychotic therapies. Augmentation typically involves the addition of a second agent to either improve therapeutic outcomes or target specific symptom domains [19]. Augmentation strategies could be categorized into pharmacological and non-pharmacological strategies. Among the pharmacological ones, the augmentation strategies may consist of the addition of a second antipsychotic agent, an antidepressant, a mood stabilizer, an anticonvulsant, or a glutamatergic agent [20]. However, in clinical practice, augmentation most commonly includes another antipsychotic, while the use of antidepressants and mood stabilizers remains less prevalent [21]. Numerous studies have investigated the efficacy of both typical and atypical antipsychotics in augmenting clozapine treatment [17, 22-24]. However, despite these efforts, no combined treatment regimen has demonstrated consistent success in enhancing the effectiveness of clozapine [25, 26].

A recent meta-analysis by Yeh *et al*. evaluating augmentation strategies for clozapine in the treatment of CRS failed to demonstrate the superiority of any of the assessed antipsychotics (quetiapine, pimozide, risperidone, sertindole, ziprasidone, aripiprazole, sulpiride, amisulpride) over placebo [27]. Regarding third-generation antipsychotics, studies examining agents like cariprazine and lurasidone are limited, with available evidence predominantly consisting of case reports and case series [28-31].

Among antipsychotic augmentation options, Lurasidone, a newer atypical agent, has attracted attention for its advantageous side effect profile and distinctive pharmacological properties. Lurasidone primarily functions as an antagonist at D2 and 5-HT2A receptors, with additional moderate antagonism at alpha2C and alpha2A adrenergic receptors [32]. Additionally, it acts as a partial agonist at 5-HT1A receptors and exhibits minimal affinity for H1 histaminergic and M1 muscarinic receptors [33]. This profile enables Lurasidone to address both positive and negative symptoms of schizophrenia. Another key advantage of this medication is its relatively low incidence of metabolic side effects [33, 34], which is crucial in managing schizophrenia, where metabolic complications are often encountered [35-37]. From a pharmacokinetic standpoint, Lurasidone undergoes extensive hepatic metabolism, and its absorption is enhanced when taken with food [38]. A thorough understanding of Lurasidone's pharmacokinetics is essential for maximizing therapeutic outcomes while minimizing potential drug interactions.

Lurasidone has shown potential in improving cognition, general psychopathology, and overall outcomes in patients with TRS [39]. Given Clozapine’s well-established efficacy in addressing the core symptoms of schizophrenia, the addition of Lurasidone may represent a promising strategy to target residual symptoms in patients already receiving Clozapine. Indeed, the combination of Clozapine and Lurasidone constitutes a novel therapeutic approach for managing CRS, leveraging the potential synergistic effects of these agents to enhance treatment outcomes [40].

Given these premises and the critical role of augmentation strategies in improving clinical outcomes in individuals with residual symptoms, a large multicenter observational study was initiated to assess and compare the efficacy of Lurasidone augmentation to Clozapine with other antipsychotic combinations. The study comprehensively examines multiple psychopathological domains, with a specific focus on resistant subtypes of schizophrenia, to evaluate the potential advantages of this strategy in managing complex and treatment-resistant cases.

## 2 MATERIALS AND METHODS

### 2.1 Characteristics of the Study

We conducted a prospective, multicenter, comparative observational study. We compared Lurasidone as an augmentation strategy in patients already treated with clozapine with other SGAs. All the subjects that met the inclusion criteria between 2021 and 2023 were included in the study and were enrolled after starting the new SGA. All the patients were observed for a six-month follow-up period. Inclusion criteria were to be diagnosed with a schizophrenia spectrum disorder according to ICD-10, to be labeled as “treatment resistant”, and to be currently under treatment with clozapine and another SGA as an augmentation strategy. There were no exclusion criteria. We accepted the definition of TRS as a clinical condition in which the symptoms persist or do not significantly improve despite the patient having received adequate treatment with two different antipsychotic medications [41]. Before collecting data, informed consent was obtained for data collection for research purposes. Data were collected anonymously by a trained psychiatrist or a psychiatry resident. Subjects were treated in various community mental health facilities located in northern Italy. The research project complied with the principles of the Declaration of Helsinki regarding medical research in humans, following research ethical requirements. Approval was granted by the University of Siena Institutional Review Board—Tuscany Area Vasta South-East Ethical Committee, prot 20068-28/6/2021.

The primary objectives were to examine the differential impact of augmentation strategies on symptomatology and functional outcomes in TRS patients receiving clozapine. Specific attention was paid to assessing the effectiveness of augmentation with Lurasidone in comparison to alternative antipsychotic augmentation regimens.

The study encompassed a diverse patient population diagnosed with TRS, confirmed through rigorous clinical assessments and adherence to standardized diagnostic criteria. Inclusion criteria ensured that all participants were actively receiving clozapine treatment, reflecting the real-world scenarios encountered in clinical practice.

### 2.2 Assessment Instruments

A battery of validated assessment tools, covering positive and negative symptoms, cognitive function, and overall psychosocial functioning, was administered at regular intervals over a comprehensive six-month observational period. Patients were assessed at T0 (initiation of the augmentation strategy), T1 (one month), and T2 (six months).

To ensure a comprehensive evaluation, clinician-rated scales, patient-reported outcomes, and objective measures were incorporated. Various scales, including the SF-12 (for quality of life), CGI (Clinical Global Impressions), PANSS (Positive and Negative Syndrome Scale), and BPRS (Brief Psychiatric Rating Scale), were employed. All the assessments were administered by a psychiatrist or a resident in psychiatry, except for the SF-12, which is a self-reported measure. All scales were applied according to the previously mentioned timeline.

Longitudinal data collection meticulously captured the dynamic evolution of symptoms and functional outcomes over the extended six-month period. Regular follow-ups and assessments, using the specified scales and measures, facilitated tracking changes in response to different augmentation strategies.

#### 2.2.1 SF-12

SF-12 is a widely used self-report measure designed to assess health-related quality of life. Derived from the longer SF-36, it consists of 12 questions covering physical and mental health domains. These questions explore various aspects such as physical functioning, role limitations due to physical and emotional problems, mental health, social functioning, pain, and general health perception. SF-12 yields two summary scores: the Physical Component Summary (PCS) and the Mental Component Summary (MCS), providing insights into the patient's overall well-being.

#### 2.2.2 Clinical Global Impressions

CGI is a clinician-rated assessment tool designed to capture an overall impression of the patient's illness severity, global improvement or deterioration, and therapeutic efficacy. It involves three subscales: Severity of Illness (CGI-S), Global Improvement (CGI-I), and Efficacy Index (CGI-E). CGI-S assesses the current severity of the patient's condition, CGI-I evaluates overall improvement or worsening, and CGI-E measures the efficacy of treatment. Widely applied in clinical trials and psychiatric research, CGI provides a clinician's holistic judgment of the patient's status.

#### 2.2.3 Positive and Negative Syndrome Scale

PANSS is a comprehensive clinical instrument developed to assess the symptomatology of schizophrenia. It consists of 30 items categorized into three subscales: the Positive Scale (PANSS-P), the Negative Scale (PANSS-N), and the General Psychopathology Scale (PANSS-G). Positive symptoms involve experiences beyond normal functioning, negative symptoms relate to deficits in normal functioning, and general psychopathology assesses the overall severity of psychopathology. PANSS is widely recognized for its reliability and validity in capturing the nuances of schizophrenia symptomatology.

#### 2.2.4 Brief Psychiatric Rating Scale

BPRS is a clinician-administered scale designed to assess a broad range of psychiatric symptoms. It comprises 18 items and covers various domains, including anxiety, depression, hallucinations, unusual behavior, and conceptual disorganization. Each item is rated on a scale from 1 to 7, with higher scores indicating greater symptom severity. BPRS is valuable in monitoring changes in symptomatology over time and evaluating treatment effectiveness in diverse psychiatric populations.

### 2.3 Statistical Analysis

Stratified analyses were conducted to explore potential variations in treatment response among distinct patient subgroups. Continuous variables were described using the median and the interquartile range (IQR). The frequency of categorical variables was expressed as the number and percentage of cases. Continuous variables were compared using the Mann-Whitney test, while the Chi-square test was used to compare categorical variables. A *p*-value of less than 0.05 was deemed to indicate statistical significance.

Missing data regarding subjects lost to follow-up were considered “Missing at Random” (MAR) and imputed using predictive mean matching in a model of “Multiple Imputation by Chained Equation” (MICE) [42].

Differences in the scores of SF-12 - considering both PCS and MCS –, PANSS-P, PANSS-N, PANSS-G, PANSS total score, CGI-S, and BPRS in T0, T1, and T2 were evaluated using the Wilcoxon signed-rank test with the Hommel *p*-value adjustment method, as well as differences in the scores of CGI-I in T1 and T2.

A linear mixed-effect regression (LMER) was used to determine if the observed differences in the scores of PCS, MCS, PANS-P, PANSS-N, PANSS-G, PANSS total score, CGI-S, CGI-I, and BPRS followed a trend dependent on the augmentation strategy with lurasidone or another atypical antipsychotic. Study variables were conceptually organized according to their role in the models. Age and gender were used to describe the sample and were also included as covariates in all analyses. As mentioned above, the primary outcomes were scores from psychometric scales, assessed longitudinally across multiple time points. The main exposure of interest was treatment type, categorized as Lurasidone *versus* other SGAs. Diagnostic category (*e.g*., schizophrenia, schizoaffective disorder) was included as a potential effect modifier. To assess whether the effect of treatment varied by diagnosis, an interaction term between treatment and diagnosis was tested within the models. Particularly, we built a model that considered the trend of psychometric evaluation over time, accounting for the effect of age, gender, diagnosis, the two different augmentation strategies, and the interactions between diagnosis and treatment and between treatment and time. Variable selection was performed using backward elimination: variables were sequentially removed based on their statistical significance until only those meeting the predefined significance criterion remained. The effect of the time was retained in all iterations to ensure its inclusion in the final model. LMER assumptions of variance homogeneity and normal distribution of the residuals were checked.

Statistical analysis was conducted using RStudio version 4.3.3.

## 3 RESULTS

The total sample consisted of 45 subjects, of whom 24 (53.33%) were treated with Lurasidone as an augmentation strategy and 21 (46.67%) with another second-generation antipsychotic (SGA). The median age was 39 years [30-50], with females comprising 31.11% of the sample. No significant differences were observed in sociodemographic or baseline clinical features, except for the mean number of psychiatric ward admissions after initiating new therapy (*p<*.05). Sociodemographic and clinical features are summarized in Table **1**. Considering the whole sample, 27 subjects (60.00%) received a diagnosis of schizophrenia, while 12 (26.67%) were affected by schizoaffective disorder, and 6 (13.33%) had other psychotic disorders. No statistically significant differences were found between the two samples regarding psychometric evaluation at T0 (Table **2**). A graphical representation of the psychometric scores over time in the whole sample is shown in Fig. (**1**), while the modification over time in the two groups is represented in Fig. (**2**).

### 3.1 PCS Score

The PCS score improved significantly over time in the entire sample, from T0 (33.29 (28.58-47.04)) to T1 (43.8 (35.44-51.08)) and T2 (48.23 (39.54-52.22)). These differences were statistically significant (*p<*.001) between T0 and subsequent timepoints, and between T1 and T2 (*p<*.05). For the Lurasidone group, the PCS scores were: T0 = 34.86 (28.93-44.17), T1 = 42.9 (33.80-50.81), T2 = 48.14 (45.46-52.22) (*p<*.01). Comparisons between T0 and subsequent timepoints (*p<*.01) and between T1 and T2 (*p<*.05) were significant. In the other SGA group, scores increased as follows: T0 = 32.15 (28.44-50.78), T1 = 44.05 (40.43-51.08), T2 = 48.23 (38.27-53.16) (*p<*.05). Differences between T0 and T1 (*p<*.01), and T0 and T2 (*p<*.05) are significant.

### 3.2 MCS Score

The MCS score also demonstrated significant improvements. In the whole sample, scores rose from T0 (28.50 (24.62-36.16)) to T1 (39.86 (32.49-46.50)) and T2 (48.22 (44.44-53.63), with all changes being statistically significant (*p<*.001). In the Lurasidone group, the MCS score increased from T0 (27.54 (24.62-31.75)) to T1 (37.48 (31.46-48.18)) and T2 (49.20 (45.02-53.63)) (*p<*.001 for all comparisons). Similarly, for the other SGA group, the scores improved from T0 (33.98 (24.90-37.43)) to T1 (40.13 (36.01-45.40)) (*p<*.01) and T2 (47.99 (44.44-52.28)) (*p<*.001).

### 3.3 CGI-S Score

The CGI-S score decreased significantly across all timepoints for the total sample: T0 (6.00 (5.00-6.00)), T1 (4.00 (4.00-5.00)), and T2 (3.00 (2.00-4.00)) (*p<*.001 for all comparisons). For the Lurasidone group: T0 = 5.00 (5.00-6.00), T1 = 4.00 (4.00-5.00), T2 = 3.00 (2.00-4.00) (*p<*.001). In the other SGA group, scores were: T0 = 6.00 (5.00-6.00), T1 = 5.00 (4.00-5.00), and T2 = 3.00 (2.00-4.00). Differences between T0 and T1 (*p<*.05), T1 and T2 (*p<*.01), and T0 and T2 (*p<*.001) were significant.

### 3.4 PANSS Score

#### 3.4.1 PANSS-P

Considering the whole sample, a reduction over time in the PANSS-P was observed (T0: 29.00 (19.00-36.00), T1: 17.00 (13.00-21.00), T2: 11.00 (9.00-15.00)), differences between T0 and T1, T0 and T2, and T1 and T2 are significant (*p<*.001). The same statistically significant differences were observed in both the Lurasidone group (T0: 28.00 (22.00-34.25), T1: 16.00 (13.00-19.50), T2: 11.00 (9.00-12.25)) (*p<*.001), and the other SGA group (T0: 30.00 (17.00-36.00), T1: 18.00 (14.00-25.00), T2: 13.00 (9.00-16.00)) (*p<*.001).

#### 3.4.2 PANSS-N

An overtime decrease in the PANSS-N was observed in the whole sample (T0: 23.00 (16.00-30.00), T1: 14.00 (9.00-21.00), T2: 9.00 (7.00-15.00)), differences between T0 and T1, T0 and T2, and T1 and T2 are significant (*p<*.001). The same statistically significant differences were observed in both the Lurasidone group (T0: 22.00 (13.75-30.00), T1: 13.00 (7.75-18.75), T2: 8.00 (7.00-14.75)) (*p<*.001) and the other SGA group (T0: 24.00 (18.00-30.00), T1: 17.00 (9.00-21.00), T2: 11.00 (8.00-15.00)) (*p<*.01).

#### 3.4.3 PANSS-G

Considering the PANSS-G, an over time decrease in the whole sample was observed (T0: 48.00 (42.00-59.00), T1: 30.00 (26.00-45.00), T2: 24.00 (20.00-34.00)), differences between T0 and T1, T0 and T2, and T1 and T2 are significant (*p<*.001). The same statistically significant differences were observed in the Lurasidone group (T0: 46.50 (41.75-57.25), T1: 29.00 (26.00-36.50), T2: 23.50 (19.75-34.25)) (*p<*.001). Considering the PANSS general psychopathology subscale score in subjects treated with another SGA (T0: 50.00 (43.00-60.00), T1: 37.00 (29.00-50.00), T2: 25.00 (22.00-30.00)), statistically significant differences between T0 and T1 (*p<*.01), T0 and T2 (*p<*.001), and T1 and T2 (*p<*.001) were observed.

#### 3.4.4 PANSS Total Score

Considering the whole sample, a reduction over time in the PANSS was observed (T0: 98.00 (87.00-116.00), T1: 59.00 (53.00-84.00), T2: 46.00 (38.00-66.00)), differences between T0 and T1, T0 and T2, and T1 and T2 are significant (*p<*.001). The same statistically significant differences were observed in the Lurasidone group (T0: 97.00 (81.75-110.75), T1: 57.00 (52.25-75.50), T2: 42.00 (37.75-66.25)) (*p<*.001). Considering the subjects treated with other SGAs (T0: 101 (89.00-120.00), T1: 71.00 (57.00-88.00), T2: 48.00 (42.00-57.00)), the differences between T0 and T1 (*p<*.01), T0 and T2 (*p<*.001), and T1 and T2 (*p<*.001) are significant.

### 3.5 BPRS Score

Regarding the BPRS score, a reduction over time was observed in the whole sample (T0: 59.00 (47.00-66.00), T1: 37.00 (32.00-46.00), T2: 26.00 (22.00-35.00)), differences between T0 and T1, T0 and T2, and T1 and T2 are significant (*p<*.001). The same statistically significant differences were observed in the Lurasidone group (T0: 55.00 (46.25-62.25), T1: 36.00 (32.00-39.00), T2: 26.00 (22.00-34.25)) (*p<*.001). Considering the BPRS score of subjects treated with other SGA (T0: 60.00 (47.00-69.00), T1: 39.00 (31.00-49.00), T2: 27.00 (26.00-35.00)), the differences between T0 and T1 (*p<*.01), T0 and T2 (*p<*.001), and T1 and T2 (*p<*.001) are significant.

### 3.6 CGI-I Score

Regarding the CGI-I score, a statistically significant reduction in T2 compared to T1 was observed in the whole sample (T1: 4.00 (3.00-4.00), T2: 3.00 (2.00-3.00)) (*p<*.001), in the Lurasidone group (T1: 3.50 (3.00-4.00), T2: 3.00 (2.00-3.25)) (*p<*.001), and in the group of subjects treated with other SGA (T1: 4.00 (3.00-5.00), T2: 3.00 (2.00-3.00)) (*p<*.01).

### 3.7 LMER Models

Three LMER models for each psychometric test were built: the first model considered just the random intercept (empty model), the second one considered the score variation over time (minimum model), and the third model considered the effect of age, gender, diagnosis, the two different augmentation strategies, and the interactions between diagnosis and treatment and between treatment and time (full model). LMER assumptions of variance homogeneity and normal distribution of the residuals were not respected for CGI-S and CGI-I scores. Variable selection was performed using backward elimination starting from the full model: variables were sequentially removed based on their statistical significance until only those meeting the predefined significance criterion remained. The effect of the time was retained in all iterations to ensure its inclusion in the final model. Selected models are summarized in Table **3**, and a graphical representation is shown in Fig. (**3**).

## 4 DISCUSSION

The results of this study provide important insights into the clinical effectiveness of lurasidone as an augmentation strategy for patients already treated with clozapine. A comparison with other SGAs suggests that both approaches led to significant clinical improvements across multiple domains. However, some differences and specific trends underscore the potential benefits of lurasidone in certain aspects of psychotic disorder management.

### 4.1 Clinical Outcomes

The significant reduction in PANSS scores across the total sample highlights the effectiveness of augmentation strategies in reducing psychotic symptoms. Importantly, the observed reductions in PANSS positive, negative, and general psychopathology subscale scores were statistically significant for both the lurasidone and other SGA groups, indicating the broad efficacy of these treatments. Moreover, the reductions in all the PANSS subscales were clinically significant, showing that an augmentation strategy could be an option for TRS patients already taking clozapine. However, data on the efficacy of augmentation of clozapine with another antipsychotic are still lacking [1, 35], indicating the need for further study. Similarly, CGI-S and CGI-I scores revealed consistent improvements over time in both groups. These findings align with the observed reductions in BPRS scores, indicating enhanced overall psychopathology management and functional outcomes. Considering the subset of subjects who received lurasidone as an augmentation strategy, a non-significant greater improvement was observed in certain domains. Particularly, the greater improvement in the PANSS positive and negative subscales could be indicative of a trend toward superior symptom control, while the larger reduction in CGI-S scores in the lurasidone group compared to the other SGAs points toward its potential to address residual symptom burden more effectively. These findings could be related to superior symptom control and reduction of psychotic relapse in people treated with lurasidone. Indeed, an intriguing observation was the significant difference in psychiatric ward admissions after introducing the augmentation strategy, with no admissions in the lurasidone group and a mean of 0.48 admissions in the other SGA group. This finding suggests that lurasidone may have an edge in preventing acute psychiatric crises requiring hospitalization, potentially attributable to its unique pharmacological profile and ability to manage symptoms more comprehensively. However, further studies are warranted to explore the underlying mechanisms of this association.

### 4.2 Functional Outcomes

The improvements in MCS and PCS scores over time provide further evidence of enhanced functional outcomes associated with both augmentation strategies. Patients receiving lurasidone showed consistent improvements across all timepoints, demonstrating its ability to improve mental well-being and physical functioning. According to an open-label trial that assessed the modification in the health-related quality of life in patients with schizophrenia who switched to lurasidone from a previous different antipsychotic, an improvement in MCS was observed after six weeks in all the patients [43]. Regarding the PCS score, differences were observed between week 6 and week 30 after the switch to lurasidone [44]. Conversely, our findings highlighted an initial positive and statistically significant effect on physical health in the first month after the new therapy introduction, while the improvement registered after six months of follow-up was not significant. It is possible that the differences found by Awad and colleagues were largely dependent on the major tolerability of lurasidone in comparison with the previous treatment. However, our findings showed that the improvement in the health-related quality of life regarding the physical component in TRS patients could be significant in the short term. Our results showed that the MCS scores were comparable between groups at the end of the study, while a slight advantage in PCS scores emerged in the lurasidone group, which may reflect its favorable tolerability profile and fewer treatment-related side effects.

### 4.3 Tolerability and Safety

The absence of significant differences in side effects, such as gastrointestinal disturbances, sedation, or tachycardia, supports the idea that both lurasidone and other SGAs were well-tolerated. This is particularly notable for lurasidone, which is known for its favorable metabolic profile. Maintaining effective symptom control without introducing significant adverse effects is critical in this patient population, where treatment adherence often remains challenging.

### 4.4 Implications for Clinical Practice

Lurasidone appears to be a viable augmentation option for patients with persistent psychotic symptoms despite clozapine treatment. Its efficacy in symptom reduction, functional improvement, and the apparent reduction in hospitalization risk may make it a particularly appealing choice for clinicians. The improvements observed across psychometric measures in both treatment groups also underscore the value of carefully selected augmentation strategies tailored to the individual patient’s needs.

Furthermore, according to the LMER results, except for the PCS score, all the functional and psychometric evaluations significantly improved after six months of follow-up, suggesting that the positive effects of the augmentation strategy could be even greater after a longer observation period.

## 5 STUDY LIMITATIONS

While the findings are promising, some limitations should be considered. Both the observational design and the relatively small sample size may have reduced the generalizability of the results. Additionally, the potential variability in adherence to prescribed augmentation regimens may have influenced the outcomes. Future studies with larger sample sizes and longer follow-up periods are needed to confirm these findings and further explore the comparative efficacy of different SGAs in this context.

## CONCLUSION

Augmentation strategies for clozapine in the treatment of CRS have been extensively studied, yet challenges remain in identifying the optimal combination to achieve significant clinical improvement. Several antipsychotics, such as Aripiprazole, Risperidone, and Amisulpride, have been explored with varying degrees of success [45, 46]. While these options demonstrate efficacy in certain domains, their variable impact on cognitive function and overall symptomatology limits their universal applicability. Lurasidone has emerged as a promising candidate due to its distinctive pharmacological profile, characterized by potent serotonin 5-HT7 and dopamine D2 receptor antagonism, moderate affinity for 5-HT2A and 5-HT1A receptors, and minimal activity at muscarinic receptors [47]. These properties suggest a potential advantage for improving cognitive deficits and mood disturbances, key domains often inadequately addressed by other antipsychotics [48]. However, in this study, no specific investigations were conducted to evaluate cognitive symptoms, an aspect that deserves particular attention given the pharmacodynamic profile of Lurasidone. Future studies should investigate whether Lurasidone, when used as an augmentation strategy, proves more effective in addressing cognitive symptoms. Compared to agents such as Aripiprazole, which primarily modulates dopaminergic activity, or Risperidone, which lacks significant pro-cognitive effects, Lurasidone demonstrates a broader therapeutic spectrum with improved tolerability, particularly concerning metabolic adverse events [49].

Additionally, specific analyses of the different dosages of lurasidone were not performed due to the small sample size, highlighting another area for investigation in future research. Indeed, a Network Meta-Analysis compared the efficacy, acceptability, and safety of ten independent treatments, which included five doses of Lurasidone (148#, 111#, 74#, 37#, and 18.5# mg/day), four antipsychotics (*i.e*., Haloperidol, Olanzapine, Quetiapine, and Risperidone), and placebo. Authors found that Lurasidone 148#, 111#, 74#, and 37# mg/day were significantly superior to placebo. Furthermore, among these four doses, Lurasidone 148# mg/day was found to be the most effective [50].

The results of this observational multicenter study suggest that Lurasidone augmentation in CRS offers significant improvements in psychopathological domains similar to alternative augmentation strategies. Its relatively favorable side-effect profile further underscores its potential utility. These findings align with recent literature emphasizing the importance of individualized augmentation strategies tailored to the specific residual symptom profile of CRS patients [45]. Nonetheless, given the aforementioned limitations, the possibility that Lurasidone may demonstrate superior efficacy compared to other augmentation strategies cannot be definitively excluded. Moreover, to robustly assess differential treatment effects and inform clinical decision-making, future research should prioritize head-to-head comparisons between Lurasidone and individual comparator agents. In addition, large-scale randomized controlled trials are needed to confirm these findings and further elucidate the mechanisms underlying the observed benefits of Lurasidone, with particular attention to cognitive domains and potential dose-dependent effects.

## Figures and Tables

**Fig. (1):**
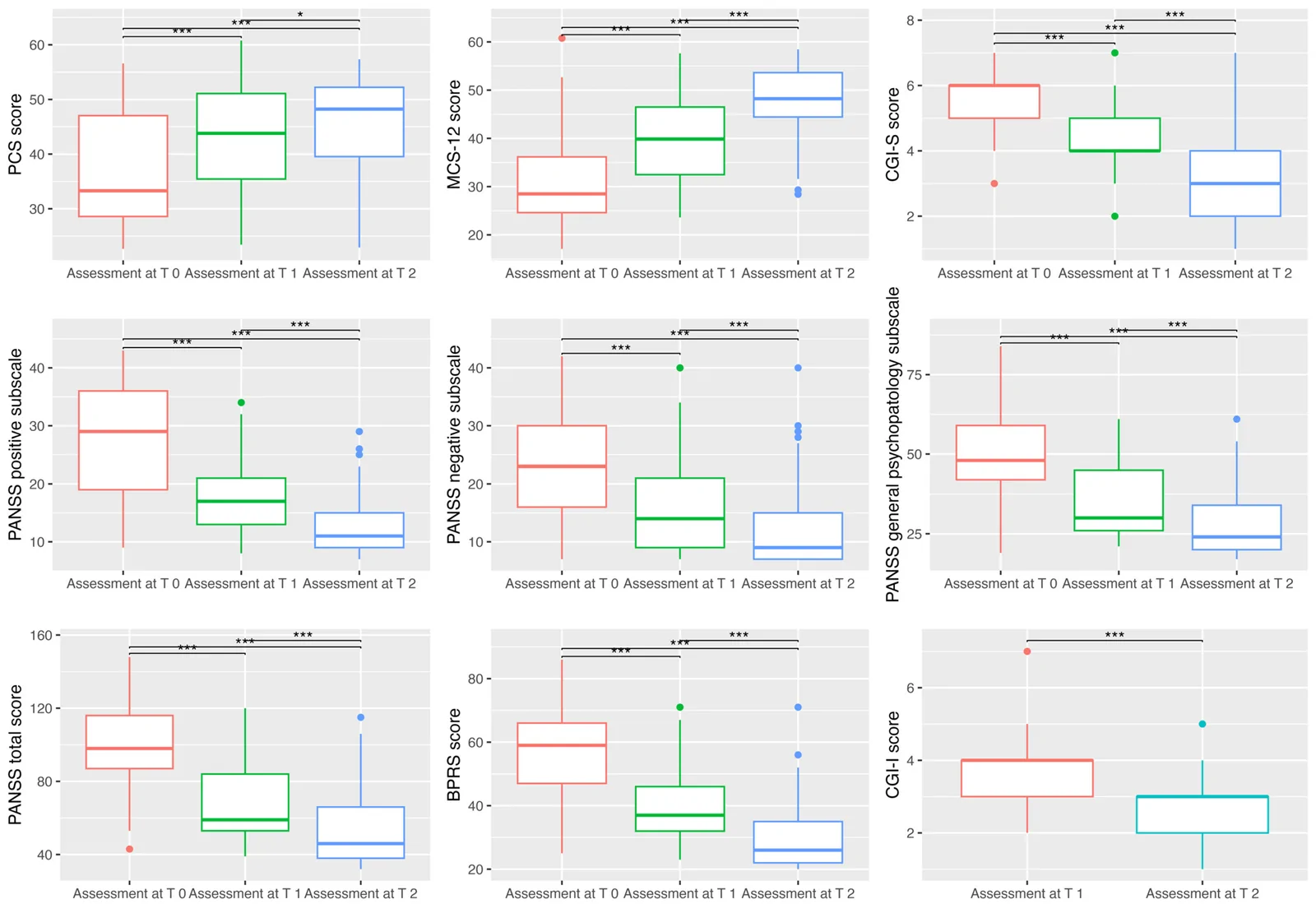
Psychometric evaluation over time in the whole sample (*p-value<.05; ***p-value<.001). PCS: Physical Component Summary of the 12-item Short Form Survey; MCS: Mental Component Summary of the 12-item Short Form Survey; CGI-S: Clinical Global Impression Severity of Illness; PANSS: Positive and Negative Syndrome Scale; BPRS: Brief Psychiatric Rating Scale; CGI-I: Clinical Global Impression Global Improvement.

**Fig. (2):**
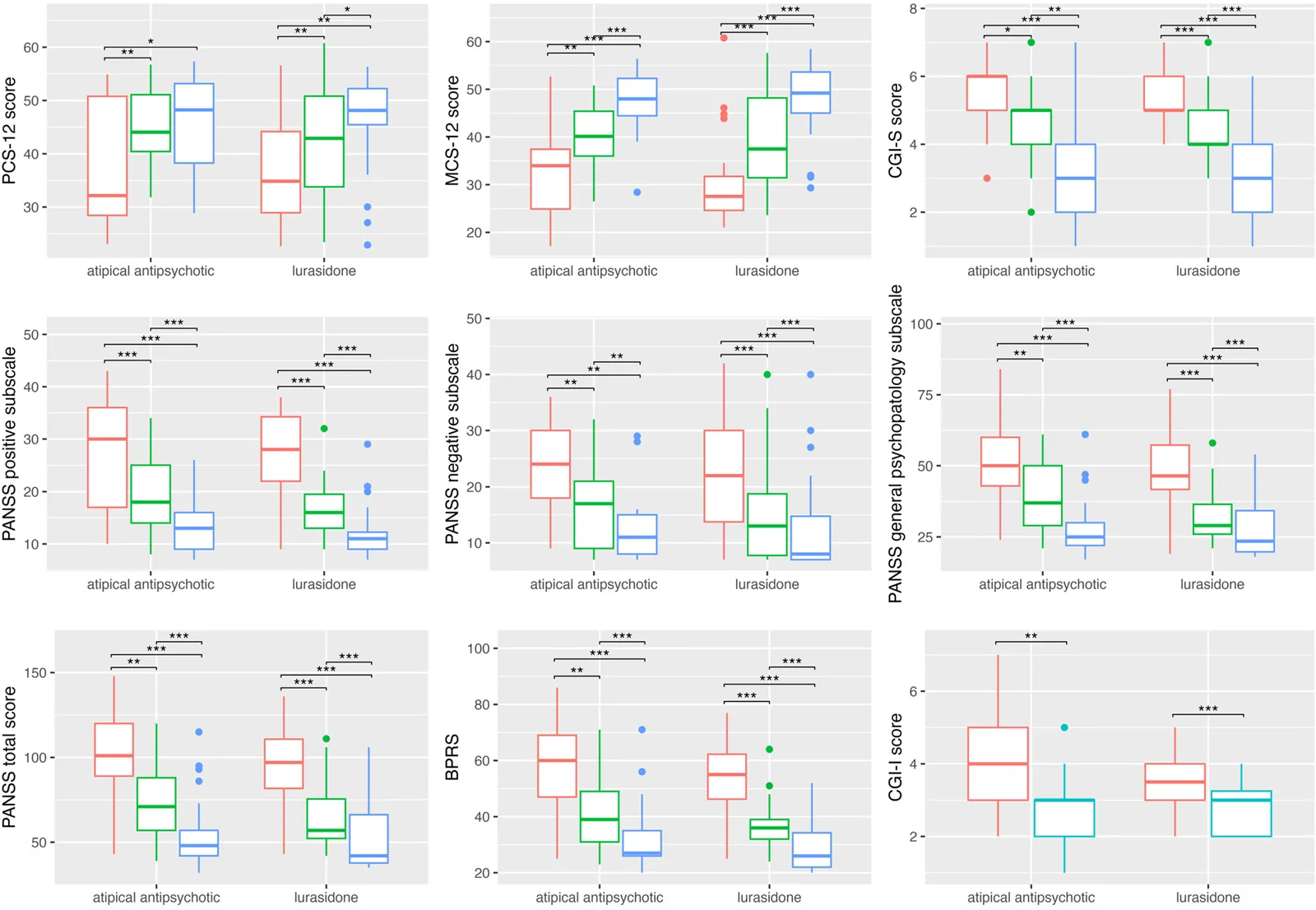
Psychometric evaluation over time in the two groups (*p-value<.05; **p-value<.01; ***p-value<.001). PCS: Physical Component Summary of the 12-item Short Form Survey; MCS: Mental Component Summary of the 12-item Short Form Survey; CGI-S: Clinical Global Impression Severity of Illness; PANSS: Positive and Negative Syndrome Scale; BPRS: Brief Psychiatric Rating Scale; CGI-I: Clinical Global Impression Global Improvement.

**Fig. (3):**
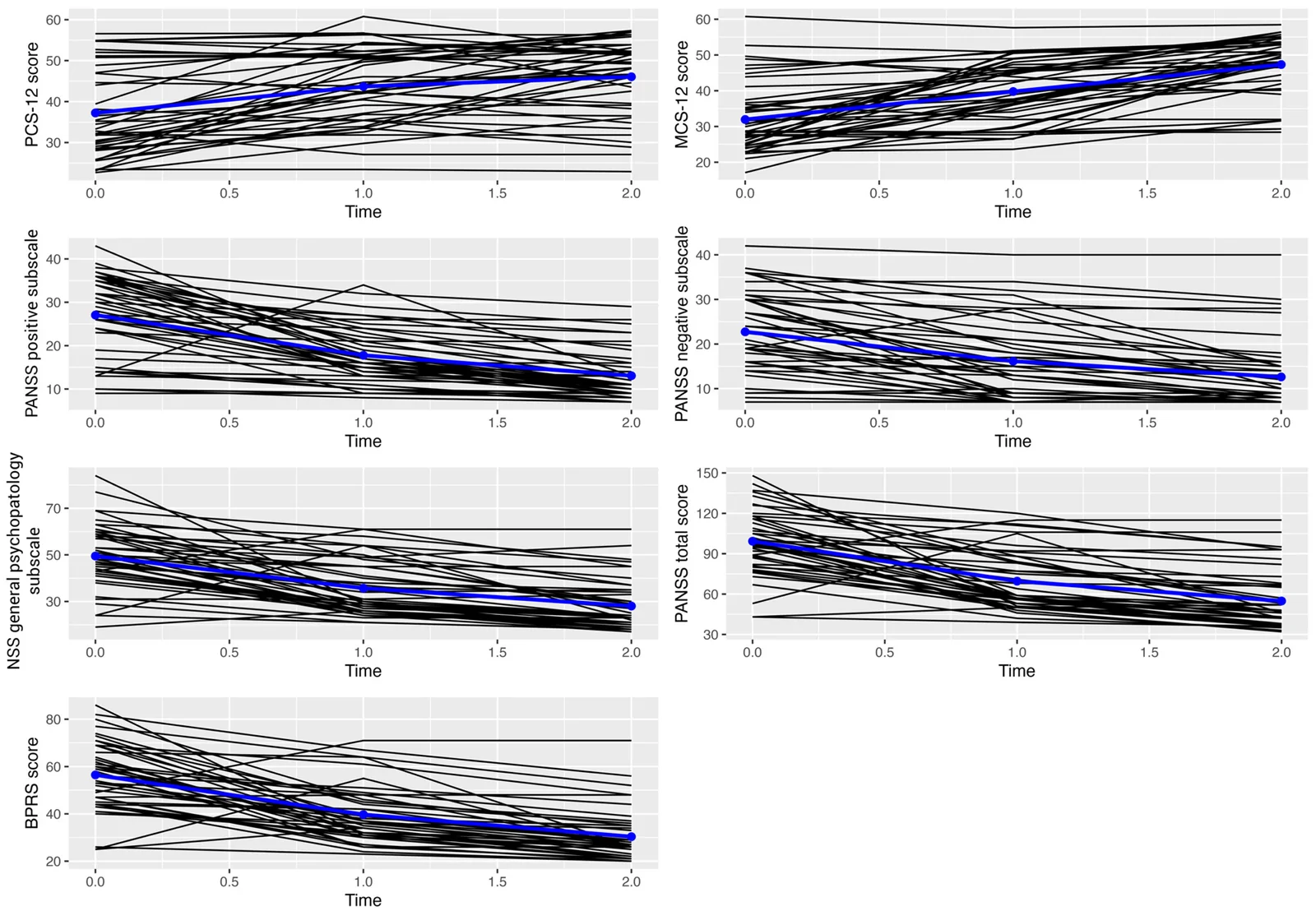
LMER models. PCS: Physical Component Summary of the 12-item Short Form Survey; MCS: Mental Component Summary of the 12-item Short Form Survey; PANSS: Positive and Negative Syndrome Scale; BPRS: Brief Psychiatric Rating Scale.

**Table 1:** Sociodemographic and clinical features.

**-**	**Overall**	**Lurasidone**	**Other SGA**	***p*-value**
N (%)	45 (100)	24 (53.33)	21 (46.67)	-
Gender (%)	-	-	-	1
Female	14 (31.11)	7 (29.17)	7 (33.33)	-
Male	31 (68.89)	17 (70.83)	14 (66.67)	-
Age (years) (median (IQR))	39 (30-50)	41.05 (30-55.25)	39 (29-47)	0.269
Educational status (less than 8 years (%))	8 (17.78)	3 (12.50)	5 (23.81)	0.549
Marital status (%)	-	-	-	0.454
Not married	38 (84.44)	20 (83.33)	18 (85.71)	-
Married	1 (2.22)	0 (0)	1 (4.76)	-
Divorced	6 (13.33)	4 (16.67)	2 (9.52)	-
Employment (%)	-	-	-	0.385
Employed	4 (8.89)	3 (12.50)	1 (4.76)	-
Unemployed	26 (57.78)	14 (58.33)	12 (57.14)	-
Registered disabled civilian	13 (28.89)	7 (29.17)	6 (28.57)	-
Student	2 (4.44)	0 (0)	2 (9.52)	-
Housing (%)	-	-	-	0.18
Lives alone	1 (2.22)	1 (4.17)	0 (0)	-
Lives with a caregiver	18 (40.00)	12 (50.00)	6 (28.57)	-
Lives in a mental health community	26 (57.78)	11 (45.83)	15 (71.43)	-
Diagnosis (%)	-	-	-	0.918
Schizophrenia	27 (60.00)	14 (58.33)	13 (61.90)	-
Schizoaffective disorder	12 (26.67)	7 (29.17)	5 (23.81)	-
Other psychotic disorder	6 (13.33)	3 (12.50)	3 (14.29)	-
Age at onset (median (IQR))	20 (19-24)	20 (19-23)	22 (20-25)	0.383
Psychiatric comorbidity (yes (%)	10 (22.22)	4 (16.67)	6 (28.57)	0.549
Type of psychiatric comorbidity (yes (%))	-	-	-	0.518
None	35 (77.78)	20 (83.33)	15 (71.43)	-
Anxiety	3 (6.67)	2 (8.33)	1 (4.76)	-
Obsessive-compulsive disorder	1 (2.22)	0 (0)	1 (4.76)	-
Personality disorder	1 (2.22)	0 (0)	1 (4.76)	-
Substance use disorder	1 (2.22)	1 (4.17)	0 (0)	-
Neurodevelopmental disorder	3 (6.67)	1 (4.17)	2 (9.52)	-
Post-traumatic stress disorder	1 (2.22)	0 (0)	1 (4.76)	-
Other medical comorbidity (yes (%))	22 (48.89)	12 (50.00)	10 (47.62)	1
Smoke (yes (%))	28 (62.22)	15 (62.50)	13 (61.90)	1
Use alcohol (yes (%))	1 (2.22)	0 (0)	1 (4.76)	0.946
Use substances (yes (%))	1 (2.22)	1 (4.17)	0 (0)	1
No compliance in the past (yes (%))	28 (62.22)	13 (54.17)	15 (71.43)	0.377
N of episodes before clozapine (more or equal than 10 (%))	10 (22.22)	4 (16.67)	6 (28.57)	0.549
Clozapine prescribed dose (mg) (median (IQR))	225 (150-400)	250 (143.75-381.25)	200 (150-400)	0.424
Clozapine dose level ng/mL (median (IQR))	297.5 (190.75-441.75)	352 (191-462)	224.8 (191.5-368)	0.480
Side effects (yes (%))	12 (26.67)	6 (25.00)	6 (28.57)	1
Gastrointestinal side effects (yes (%))	7 (15.56)	3 (12.50)	4 (19.05)	0.847
Sialorrhoea (yes (%))	5 (11.11)	1 (4.17)	4 (19.05)	0.267
Sedation (yes (%))	5 (11.11)	3 (12.50)	2 (9.52)	1
Tachycardia/Hypotension (yes (%))	1 (2.22)	0 (0)	1 (4.76)	0.946
Muscle stiffness (yes (%))	1 (2.22)	0 (0)	1 (4.76)	0.946
Seizures (yes (%))	1 (2.22)	1 (4.17)	0 (0)	1
Aspiration pneumonia (yes (%))	1 (2.22)	1 (4.17)	0 (0)	1
Time since clozapine introduction (years) (median (IQR))	3 (1-8)	5.5 (1.75-9)	2 (1-5)	0.156
N of psychiatric admissions after clozapine introduction (median (IQR))	1 (0-2)	1 (0-2)	0 (0-2)	0.557
Reason for augmentation (%)	-	-	-	0.696
Positive or negative symptoms	29 (70.73)	14 (63.64)	15 (78.95)	-
Side effects	7 (17.07)	5 (22.73)	2 (10.53)	-
Cognitive symptoms	3 (7.32)	2 (9.09)	1 (5.26)	-
Compliance	2 (4.88)	1 (4.55)	1 (5.26)	-
Setting augmentation (%)	-	-	-	0.435
Psychiatric ward	22 (48.89)	10 (41.67)	12 (57.14)	-
Outpatient clinic	10 (22.22)	7 (29.17)	3 (14.29)	-
Psychiatric community	13 (28.89)	7 (29.17)	6 (28.57)	-
N of psychiatric admission after augmentation (median (IQR))	0 (0-0)	0 (0-0)	0 (0-0)	0.012
Other medication (yes (%))	32 (71.11)	17 (70.83)	15 (71.43)	1
Assuming antdepressant (yes (%))	5 (11.11)	2 (8.33)	3 (14.29)	0.874
Assuming FGA (yes (%))	1 (2.22)	0 (0)	1 (4.76)	0.946
Assuming benzodiazepines (yes (%))	18 (40.00)	8 (33.33)	10 (47.62)	0.502
Assuming mood stabilizer (yes (%))	21 (46.67)	12 (50.00)	9 (42.86)	0.857
Other medical pharmacotherapy (yes (%))	1 (2.22)	0 (0)	1 (4.76)	0.946

**Table 2:** Psychometric evaluation at T0.

**-**	**Overall**	**Lurasidone**	**Other SGA**	**p-value**
N (%)	45 (100)	24 (53.33)	21 (46.67)	-
SF-12 PCS (median (IQR))	33.29 (28.58-47.04)	34.86 (28.93-44.17)	32.15 (28.44-50.78)	0.973
SF-12 MCS (median (IQR))	28.50 (24.62-36.16)	27.54 (24.62-31.75)	33.98 (24.90-37.43)	0.155
CGI-S (median (IQR))	6.00 (5.00-6.00)	5.00 (5.00-6.00)	6.00 (5.00-6.00)	0.567
PANSS positive symptoms subscale (median (IQR))	29.00 (19.00-36.00)	28.00 (22.00-34.25)	30.00 (17.00-36.00)	0.568
PANSS negative symptoms subscale (median (IQR))	23.00 (16.00-30.00)	22.00 (13.75-30.00)	24.00 (18.00-30.00)	0.356
PANSS general psychopathology (median (IQR))	48.00 (42.00-59.00)	46.50 (41.75-57.25)	50.00 (43.00-60.00)	0.426
PANSS total score (median (IQR))	98.00 (87.00-116.00)	97.00 (81.75-110.75)	101.00 (89.00-120.00)	0.351
BPRS (median (IQR))	59.00 (47.00, 66.00)	55.00 (46.25, 62.25)	60.00 (47.00, 69.00)	0.322

**Note**: SF-12: 12-item Short Form Survey; PCS: Physical Component Summary; MCS: Mental Component Summary; CGI-S: Clinical Global Impression Severity of Illness; PANSS: Positive and Negative Syndrome Scale; BPRS: Brief Psychiatric Rating Scale.

**Table 3:** LMER selected models.

**-**	**Estimate**	**95% Confidence Interval**		***p*-value**
**SF-12 PCS**	*Score ~ D1 + D2 + (1 | ID)*			
(Intercept)	37.232	34.406	40.057	≤0.001
D1	6.456	3.937	8.976	≤0.001
D2	2.368	-0.152	4.888	0.069
**SF-12 MCS**	*Score ~ D1 + D2 + (1 | ID)*			
(Intercept)	31.958	29.380	34.536	≤0.001
D1	7.815	5.161	10.469	≤0.001
D2	7.541	4.887	10.195	≤0.001
**PANNS Positive Subscale**	*Score ~ D1 + D2 + (1 | ID)*			
(Intercept)	27.044	24.908	29.181	≤0.001
D1	-9.267	-11.456	-7.077	≤0.001
D2	-4.711	-6.901	-2.522	≤0.001
**PANNS Negative Subscale**	*Score ~ D1 + D2 + (1 | ID)*			
(Intercept)	22.733	20.258	25.209	≤0.001
D1	-6.578	-8.400	-4.756	≤0.001
D2	-3.533	-5.356	-1.711	≤0.001
**PANNS General Psychopathology Subscale**	*Score ~ D1 + D2 + (1 | ID)*			
(Intercept)	49.489	45.973	53.005	≤0.001
D1	-13.800	-17.406	-10.194	≤0.001
D2	-7.644	-11.250	-4.038	≤0.001
**PANNS Total Score**	*Score ~ D1 + D2 + (1 | ID)*			
(Intercept)	99.267	92.644	105.889	≤0.001
D1	-29.667	-36.355	-22.978	≤0.001
D2	-14.733	-21.422	-8.045	≤0.001
**BPRS**	*Score ~ D1 + D2 + (1 | ID)*			
(Intercept)	56.467	52.785	60.148	≤0.001
D1	-16.756	-20.767	-12.744	≤0.001
D2	-9.356	-13.367	-5.344	≤0.001

**Note**: D1 = 0 in T0, D1 = 1 in T1 and T2; D2 = 0 in T0 and T1, D2 = 1 in T2.

SF-12: 12-item Short Form Survey; PCS: Physical Component Summary; MCS: Mental Component Summary; PANSS: Positive and Negative Syndrome Scale; BPRS: Brief Psychiatric Rating Scale.

## Data Availability

The data presented in this study are available on request from the corresponding author due to privacy reasons.

## LIST OF ABBREVIATIONS

BPRS: Brief Psychiatric Rating Scale
CGI-E: Clinical Global Impression – Efficacy Index
CGI-I: Clinical Global Impression – Improvement
CGI-S: Clinical Global Impression – Severity of Illness
CRS: Clozapine-resistant schizophrenia
IQR: Interquartile range
LMER: Linear Mixed-Effect Regression
MAR: Missing at Random
MCS: Mental Component Summary
MICE: Multiple Imputation by Chained Equation
PANSS-G: Positive and Negative Syndrome Scale – General Psychopathology
PANSS-N: Positive and Negative Syndrome Scale – Negative Scale
PANSS-P: Positive and Negative Syndrome Scale – Positive Scale
PCS: Physical Component Summary
SGA: Second-Generation Antipsychotic
TRS: Treatment-Resistant Schizophrenia
